# Comparison of nerve combing and percutaneous radiofrequency thermocoagulation in the treatment for idiopathic trigeminal neuralgia^☆^

**DOI:** 10.1016/j.bjorl.2016.09.002

**Published:** 2016-09-27

**Authors:** Yuanfeng Du, Wenhua Yu

**Affiliations:** Nanjing Medical University Affiliated Hangzhou Hospital, The Hangzhou First People's Hospital, Department of Neurosurgery, Hangzhou, China

Dear Editor,

We read with great interest the article by Zhou et al.^1^

Zhou and colleagues compared the efficacy between nerve combing (NC) and percutaneous radiofrequency thermocoagulation (RF) in the treatment for idiopathic trigeminal neuralgia. In the group NC, the rate of satisfactory relief was 82%, satisfactory relief was 76.4% in the group RF; there were no significant differences between the two groups. As known, microvascular decompression (MVD) is the best surgical treatment for trigeminal neuralgia in patients of any age,^2^ only when the neurovascular compression (NVC) is absent, nerve combing or internal neurolysis may be considered.^3^

In this study, the authors performed nerve combing procedure in 55 cases, but they did not mention whether NVC was present or how many patients present NVC. If typical vascular compression was found at surgery, MVD was the only procedure; NV might lead to facial numbness after operation. MVD combined with NV may be used in the patients with atypical vascular compression. So the procedure and conclusion might be inaccurate without excluding NVC.

## Conflicts of interest

The authors declare no conflicts of interest.
